# How fast is fast? Eco‐evolutionary dynamics and rates of change in populations and phenotypes

**DOI:** 10.1002/ece3.1899

**Published:** 2016-01-09

**Authors:** John P. DeLong, Valery E. Forbes, Nika Galic, Jean P. Gibert, Robert G. Laport, Joseph S. Phillips, Janna M. Vavra

**Affiliations:** ^1^ School of Biological SciencesUniversity of Nebraska – LincolnLincolnNebraska68588

**Keywords:** Eco‐evolutionary dynamics, evolutionary constraint, evolutionary rate, population dynamics, rapid evolution, Taylor's power law

## Abstract

It is increasingly recognized that evolution may occur in ecological time. It is not clear, however, how fast evolution – or phenotypic change more generally – may be in comparison with the associated ecology, or whether systems with fast ecological dynamics generally have relatively fast rates of phenotypic change. We developed a new dataset on standardized rates of change in population size and phenotypic traits for a wide range of species and taxonomic groups. We show that rates of change in phenotypes are generally no more than 2/3, and on average about 1/4, the concurrent rates of change in population size. There was no relationship between rates of population change and rates of phenotypic change across systems. We also found that the variance of both phenotypic and ecological rates increased with the mean across studies following a power law with an exponent of two, while temporal variation in phenotypic rates was lower than in ecological rates. Our results are consistent with the view that ecology and evolution may occur at similar time scales, but clarify that only rarely do populations change as fast in traits as they do in abundance.

## Introduction

Understanding the pace of evolutionary change is a major objective in biology (Simpson 1944; Eldredge and Gould 1972; Kinnison and Hendry 2001). A core proposition of the burgeoning field of eco‐evolutionary dynamics is that evolutionary change is fast enough that the resulting changes in phenotype can feed back to ecological dynamics as they unfold (Thompson 1998; Yoshida et al. 2003; Fussmann et al. 2007; Palkovacs and Hendry 2010; Schoener 2011; Reznick 2013). Ignoring the influence of evolution on ecological dynamics could thus result in a critical misunderstanding of the factors responsible for population persistence, with potentially detrimental consequences for species conservation and management (Hairston et al. 2005; Kinnison and Hairston 2007; Carlson et al. 2014). Moreover, evolution may alter parameter space and generate changes in dynamic patterns that are unpredictable from standard population models (Roughgarden 1971; Fussmann et al. 2003; Yoshida et al. 2003; Otto and Day 2007). It is thus essential to determine how fast rates of evolution, or rates of phenotypic change more generally, are and how these compare with the associated rates of ecological change.

Because evolution is a population‐level process, we focus here on changes in mean traits along with changes in population size, although evolution also may be linked to other ecological processes such as metapopulation dynamics or ecosystem function (Hanski 2011; Walsh et al. 2012). To investigate the link between rates of population and evolutionary change, we begin by modifying a standard model describing the rate of directional change in the mean of trait *z*:

(1) $$\frac{dz}{dt}=h^{2}v^{2}\frac{\partial W}{\partial z},$$

where *h* ^2^ is the narrow‐sense heritability, *v* ^2^ is the additive genetic variance in the trait, *W* is mean fitness, and (∂W/∂z) is the fitness gradient (Lande 1976; Abrams et al. 1993). To look at how close this rate of evolutionary change is to the underlying change in abundance, we divide both sides of eqn (1) by *W* and substitute a standard definition of fitness (Lande 1976; Abrams et al. 1993), *W* = (1/*N*) (d*N*/d*t*), which is the per capita rate of growth. Rearranging, we obtain

(2) $$\frac{dz}{dt}\;=\left[h^{2}v^{2}\frac{\partial \;\text{log}W}{\partial z}\right]\frac{1}{N}\frac{dN}{dt}.$$

Equation (2) shows that the fraction of heritable variation, *h* ^2^ *v* ^2^, and the relative fitness gradient (∂log*W*)/∂z) are what determine how closely rates of evolutionary change may get to the associated rate of population change. In this study, we will compare these rates across species where traits vary in magnitude and dimension, so we further divide both sides of eqn (2) by *z* so that both ecological and evolutionary rates have comparable units (*t* ^−1^):

(3) $$\frac{1}{z}\frac{dz}{dt}=\left[\frac{h^{2}v^{2}}{z}\frac{\partial \;\text{log}W}{\partial z}\right]\frac{1}{N}\frac{dN}{dt}.$$

Equation (3) is an explicit directional selection framework. It does not deal with frequency‐dependent or fluctuating selection (Lande 1976), unless the fitness gradient can be linked to the ecological context. However, it does not make any further assumptions with respect to the dependency between fitness and population size, in such a way that (1/*N*) (d*N*/d*t*) can be generated by a variety of other ecological and evolutionary processes, including density dependence. This makes it a useful framework in which to consider the link between rates of phenotypic and population change across a wide range of settings.

In principle, rates of phenotypic change may be slower than, similar to, or faster than rates of change in population size. Equation (2) shows that rates of evolutionary change may be smaller than the associated rate of ecological change when the fraction of heritable variation is low or when the relative fitness gradient is shallow. Thus, even when selection is strong, low heritability, limited variance, and pleiotropy could all limit how quickly phenotypes change (Williams 1957; Barton and Partridge 2000; Futuyma 2010). In contrast, “soft” selection may allow traits to change in populations that are relatively stable in abundance (Wallace 1975), generating phenotypic change that is fast relative to the change in population size. Similarly, cryptic dynamics may generate relatively stable population sizes even when the frequency of individuals with specific alleles – and thus traits – is changing rapidly (Yoshida et al. 2007).

Another manner by which traits may change along with population size is through phenotypic plasticity (Abrams and Matsuda 2004; DeLong et al. 2014; Fischer et al. 2014). Although there may be limits on plasticity (DeWitt et al. 1998), plasticity may allow trait changes that are not limited by the fraction of heritable variation. Equation (3) does not account for phenotypic plasticity, and we do not know how or whether rates of change through plasticity should be linked to rates of change in population size. But if changes in phenotypes are additive with genetic change, rather than occurring in place of genetic change, one might predict that overall rates of phenotypic change would be faster where plasticity occurs.

Previous theory on density‐dependent selection suggests that rates of change in population size and allele frequencies should be linked, such that faster population dynamics may be associated with faster rates of trait change (Roughgarden 1971; Otto and Day 2007). Moreover, there are several clear cases of rapid evolution associated with dramatic changes in population size (Grant and Grant 2002; Fussmann et al. 2003, 2007; Hairston et al. 2005). Yet it remains unclear how rates of phenotypic change generally compare with rates of population size change.

In this study, we analyze a new data set on rates of phenotypic and population change to determine how similar these two types of rates actually are. The data set is a compilation of concurrent measurements of population size and trait change through time taken from published studies on a wide range of organisms. These rates are standardized per trait unit and generation to facilitate a comparative analysis. We focus on morphological and life‐history traits that may change through shifts in allele frequencies or cross‐generational plasticity and not traits that are behaviorally plastic. We specifically address three questions: (1) How fast are rates of phenotypic change in comparison with the associated rates of population change? (2) Do phenotypically plastic traits show relatively fast rates of change? and (3) Are systems that are relatively fast in terms of population change also relatively fast in terms of trait change? Our results clarify the nature of the relationship between evolutionary and ecological rates of change and suggest that rates of change in phenotypes should generally be slower than the associated rates of population change.

## Methods

### Data collection

We searched for studies that reported concurrent changes in phenotypes and population size. Recent reviews, compilations of evolutionary rates, and special issues of journals on eco‐evolutionary dynamics provided sources. We also searched Google scholar, specific journals, and the websites of individuals with a record of work on rapid evolution. Our search revealed 15 studies with 21 cases of temporally concurrent data on phenotypic and population change (Table 1). The taxa included algae, protists, rotifers, lizards, fish, mammals (including humans), and birds (available as Supporting information). Most of the studies were field based, but several, particularly those focused on plankton, were conducted in the laboratory. Most of the studies focused on changes in a body size dimension such as cell volume, body mass, and wing, horn, beak, or total length (Table 1). Observations on individual‐level traits were used, excluding traits that were model‐simulated rather than measured (e.g., Duffy et al. 2009). We did not use traits that can vary rapidly within the lifetime of an individual (e.g., behavioral or physiological plasticity), but did include traits that may show developmental plasticity across generations. Some studies reported changes in more than one trait (Table 1). One study reported abundance and body length for two sites within the same population for males and females separately; these data were pooled across sites and sexes (Edeline et al. 2008). We will refer to the rates of change in traits as rates of phenotypic change rather than evolutionary change because it is not always clear to what degree the changes were genetic rather than arising from phenotypic plasticity. Ecological rates were based on changes in the population abundance, density, or other indicator of population size such as number of nests.

**Table 1:** Studies used in this analysis. Mode of change is the dominant mechanism of phenotypic change

Species	Trait	Taxa	Mode	Habitat	Trans.	Location	# gens
*Brachionus calyciflorus* (Fussmann et al. 2003)	Propensity for mixis	R	G	A	M	L	22.8
*Didinium nasutum* (DeLong et al. 2014)	Cell size	P	P	A	U	L	11.5
*Cafeteria* sp. (González et al. 1993)	Cell size	P	P	A	U	L	788.4
*Marmota flaviventris* (Ozgul et al. 2010)	Body mass	M	P	T	M	F	30.0
*Petrochelidon pyrrhonota* (Brown and Brown 2013)	Wing length	B	G	T	M	F	29.1
*Ovis canadensis* (Coltman et al. 2003)	Body mass Horn length	M	G	T	M	F	5.0
*Homo sapiens* (Milot et al. 2011)	Age first reproduction	M	G	T	M	F	10.3
*Chlamydomonas reinhardtii* (Becks et al. 2012)	Cell clump size	A	G	A	U	L	145.7
*Uta stansburiana* (Sinervo et al. 2000)	Clutch size Egg mass	L	G	T	M	F	10.0
*Paraphysomonas imperforata* (Caron et al. 1985)	Cell size	P	P	A	U	L	11.2
*Anolis sagrei* (Schoener et al. 2002)	Hindlimb length # of lamellae	L	U	T	M	F	1.3
*Gadus morhua* (Swain et al. 2007)	Length	F	G	A	M	F	5.5
*Ovis aries* (Ozgul et al. 2009), (Ezard et al. 2009)	Mass	M	P	T	M	F	12.0
*Geospiza fortis* (Grant and Grant 2002)	Bill depth Bill length	B	G	T	M	F	8.0
*Strombidium sulcatum* (Fenchel and Jonsson 1988)	Cell volume	P	P	A	U	L	7.6
*Zootoca (Lacerta) oviparis* (Galliard et al. 2005)	Snout‐vent length	L	U	T	M	F	0.3
*Perca fluviatilis* (Edeline et al. 2008)	Length	F	G	A	M	F	16.7

Abbreviations are for taxa: R = rotifer, P = protist, B = bird, M = mammal, A = algae, L = lizard, F = fish; for mode of change: G = genetic, P = plastic, and U = unknown; for habitat: A = aquatic and T = terrestrial; for evolutionary transition (Trans.): M = metazoan, and U = unicell; and for location: L = laboratory and F = field.

The approximate number of generations in the time series is given.

### Data analysis

We digitized data from figures and calculated proportional rates of change in the same way for both the phenotypes and the population sizes. Observations were averaged across replicates or points near in time to match time steps between the trait and the abundance data. The rate of phenotypic change was per unit per generation *g*, calculated as: $\frac{1}{z}\frac{dz}{dg}=\frac{t_{g}}{z_{1}}\frac{z_{2}-z_{1}}{t_{2}-t_{1}}$, where *z* is the trait and *t* is time, subscripted for time 1 and time 2, and *t*_g_ is time per generation. The rate of population change was calculated per capita per generation, as: $\frac{1}{n}\frac{dn}{dg}=\frac{t_{g}}{n_{1}}\frac{n_{2}-n_{1}}{t_{2}-t_{1}}$, where *n* is the abundance or density of individuals or nests. These calculations transformed both the phenotypic and population rates of change to per unit rates per generation, which is a standardized metric that allows comparisons across species, traits, and different rates. This standardization also eliminates any concern that our results are sensitive to the time frame of sampling (Gingerich 1983, 2001). The average (±SE) rate of the absolute value of the phenotypic and population change was then calculated over the period of the study, and the variance of each rate was calculated as the variance across all time steps for each study.

## Results

**Figure 1:**
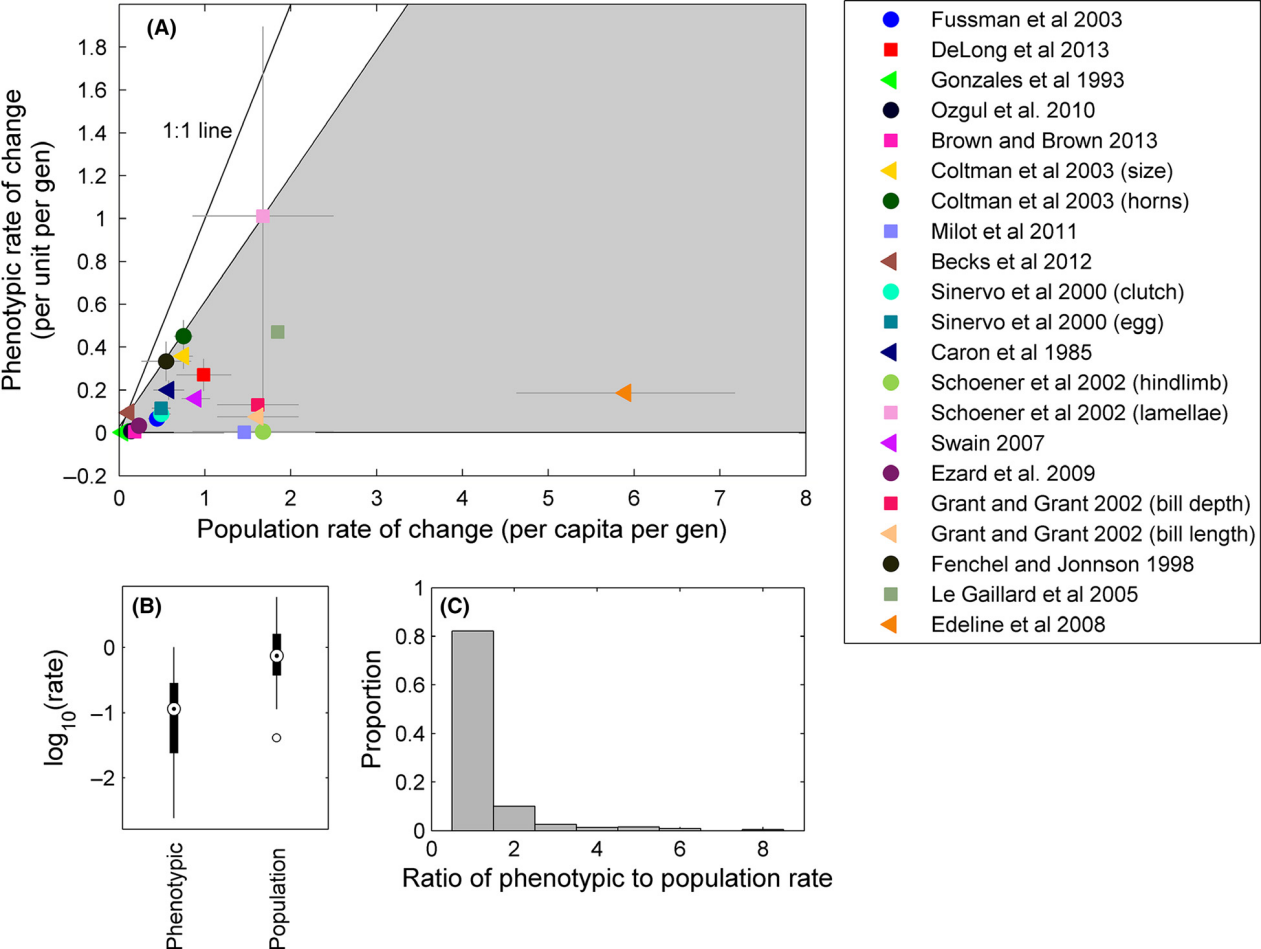
Differences between the average rates of phenotypic and population change for each study. The average is taken on the absolute value for the rate at each time step in the study. (A) The average rates plotted against each other, color and symbol coded by study and trait. The gray bars show standard error of the mean in both directions. The observations across a wide range of taxa are well defined by a constraint space set by 5% and 95% quantile regressions (gray area). (B) The mean phenotypic rate of change is significantly smaller than the mean rate of population change. (C) The ratio of phenotypic to population change within studies for each time step where the population size and trait could be time‐matched. This distribution shows that the vast majority of time steps (>82%) show faster change in population size than in phenotype. The long tail indicates that on rare occasions, trait changes were faster than population size changes. The figure excludes five instances of ratios greater than nine for clarity.

Plotted with the standardized rate of population change on the *x*‐axis and the standardized rate of phenotypic change on the *y*‐axis, our data reveal a clear constraint space demarcated with two quantile regressions: a 5% quantile regression where the slope is not different from 0 (95% CIs = −0.002 to 0.03) and a 95% quantile regression with a slope of 0.59 (CIs = 0.49–0.64) (Fig. 1A). All the points occur below a 1:1 line, indicating that the average rate of phenotypic change is less than the average rate of population change (Fig. 1A), and this is confirmed by a *t*‐test comparing the two types of rates (*t *=* *3.13; df = 40; *P *=* *0.003; Fig. 1B). A linear regression indicates there is no relationship between the rates of phenotypic change and the rates of change in population size across systems (*P *=* *0.41). The ratio of the mean rate of phenotypic to population change had a mean of 0.25 (±0.05 SE). More than 82% of individual time steps within studies showed slower rates of phenotypic than population change, but this distribution had a fat tail, indicating that at rare times, traits may be moving very quickly compared with the rate of change in the population (Fig. 1C).

The data set includes species that varied in key aspects, including aquatic or terrestrial organisms (difference in habitat), unicellular or multicellular organisms (species separated by an evolutionary transition), organisms whose mode of change involved genetic change or phenotypic plasticity, and in laboratory or field settings (Table 1). Using a GLM (with Gaussian error) with phenotypic rate as a dependent variable and population rate as a predictor, none of these factors had a significant effect after controlling for the effect of population rate of change (Fig. 3; habitat: *t *=* *0.50, *P *=* *0.62; evolutionary transition: *t *=* *0.08, *P *=* *0.94; mode of change, genetic versus plastic: *t *=* *0.08, *P *=* *0.94; setting: *t *=* *−0.13, *P *=* *0. 90). With only 21 studies, these GLM analyses had low power. The number of studies needed to achieve a power of 0.8 would be 32, 38, 120, and 330 for evolutionary transition, mode of change, habitat, and setting, respectively. This result suggests it is plausible that in the future we could detect a difference in the relative rate of phenotypic change between unicellular and multicellular organisms and between populations changing plastically or genetically. For now, however, a study's location within the gray area in Fig. 1A is not well‐predicted by these major dichotomies.

Both phenotypic and population rates displayed an increase in the temporal variance with the mean rate (Fig. 3). An ordinary least squares regression on the log‐transformed mean and variance of the rates revealed that both phenotypic and population rates followed the same power law with an exponent of ~2 (population rates: *R* ^2^ = 0.89, *P *<* *0.001; phenotypic rates: *R* ^2^ = 0.96, *P *<* *0.001).

## Discussion

In principle, rates of phenotypic change may be slower than, similar to, or faster than rates of change in population size. Our results for a wide range of taxa indicate that, excluding behaviorally plastic traits, rates of change for traits are up to about two‐third and on average about one‐fourth the associated rates of change in population size (Fig. 1). Although these differences certainly can be interpreted as being small enough to support the notion that evolution and ecology occur on the same time scales, our analysis is the first to broadly clarify that rates of phenotypic change are slower than rates of population change even when traits are changing very quickly.

Our main analysis focused on mean overall rates of change for phenotypes and population size. A similar result emerged when evaluating changes within studies at individual time steps: More than 82% of rates of phenotypic change were slower than the change in population size occurring at that time (Fig. 1C). The fat tail of this distribution suggests that on rare occasions, traits were changing very quickly with respect to changes in population size, which is consistent with the observations on the temporal distribution of selection gradients (Siepielski et al. 2009). Unless the amount of heritable variation is changing through time, eqn (3) indicates that fitness gradients are varying through time, often relatively shallow but occasionally very steep.

**Figure 2:**
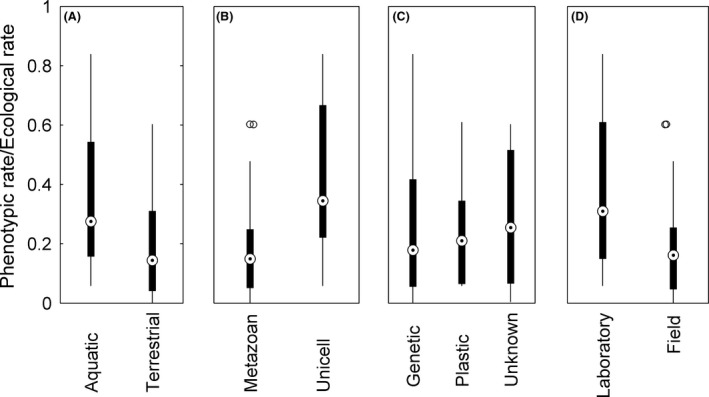
The relative speed of phenotypic and population change did not differ across major factors that differed among studies.

In three of the four cases where two traits were paired with the same rate of population change – beak length and depth in finches (Price and Grant 1984), body mass and horn length in bighorn sheep (Coltman et al. 2003), clutch size and egg mass in lizards (Sinervo et al. 2000) – the rates of phenotypic change were nearly identical. In contrast, lamellae number and hindlimb length in lizards (Schoener et al. 2002) showed different rates of phenotypic change for the same ecological rate of population change. Equation (3) suggests this is due to differences in fitness gradients or the amount of heritable genetic variation between the two traits (Lande 1976; Abrams et al. 1993). Phenotypic plasticity also could be important in setting the relationship between phenotypic and population rates, but exactly how phenotypic plasticity is linked to changes in population abundance is not clear. Nonetheless, the absence of a difference between observations where phenotypic plasticity was or was not important (Fig. 2) suggests that the mode of change (genetic vs. plastic) is not the primary driver of where points fall within the constraint space in Fig. 1A, although this conclusion may change when more studies become available.

The result that phenotypic rates of change are slower than rates of population change does not mean that observed rates of phenotypic change are unimportant with respect to eco‐evolutionary dynamics in these systems. Indeed, a landmark study on rapid evolution in the medium ground finch (Grant and Grant 2002) showed a relatively slow rate of phenotypic change compared with the rate of population change, but these changes were clearly as vital to the persistence of the species as the continuing changes in island productivity (Hairston et al. 2005). Only one study showed a rate of phenotypic change that was nearly equal to that of the rate of population change (Becks et al. 2012). In that study, the trait measured, cell clump size in the algae *Chlamydomonas reinhardtii* exposed to temporally varying levels of predation risk, is an extended phenotype (a trait that is a consequence of an individual phenotype but occurs outside of the individual's body [Dawkins 1999]). The size of the clumps can continue to increase even when allele frequencies no longer change, potentially allowing the trait's rate of change to be decoupled from the rate of change in individuals.

**Figure 3:**
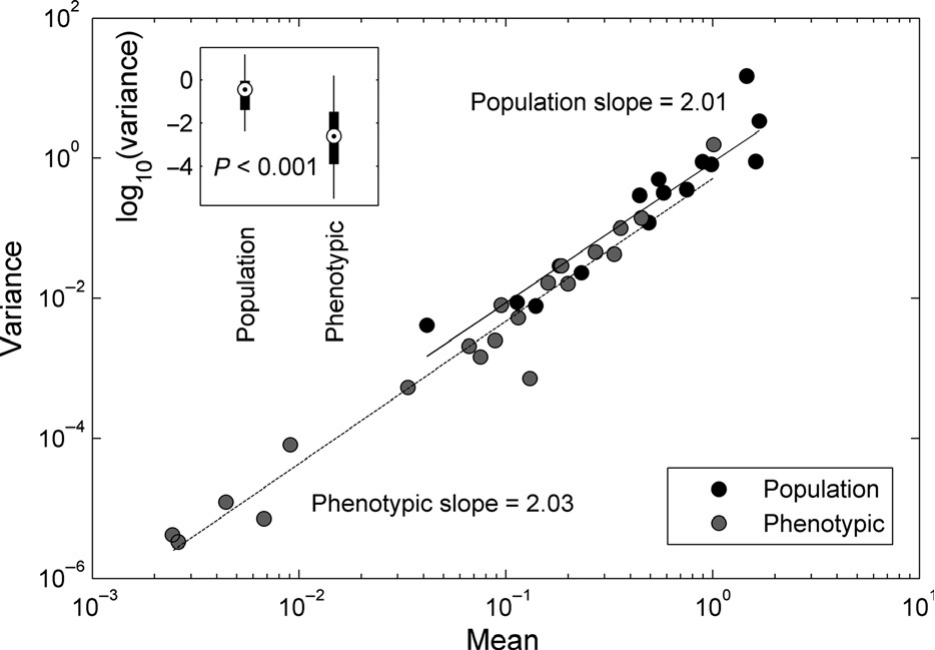
The temporal variance in the per unit rates of change for both phenotypes and populations increases with the mean across studies with a power law with an exponent of two. The variance in phenotypic rates of change was less than the variance in the rates of population change (inset).

Phenotypic rates showed lower variance than population rates (Fig. 3, inset). We speculate that this could be a consequence of phenotypes moving toward peaks in the fitness landscape while abundances range more widely. For example, body size appears to respond to optimality processes (Roff 1986; DeLong et al. 2014) but is limited by physical constraints (e.g., it cannot fall to zero). While there are certainly limits to variation in population abundance, there is evidence that many populations show larger variation than would be expected under strong regulation (Ziebarth et al. 2010).

A power law relationship between the mean and variance of a rate (known as Taylor's power law) is common for population abundance data (Taylor 1961; Kilpatrick and Ives 2003), but it is not known for rates of phenotypic change or evolution. The existence of Taylor's power law for rates of phenotypic change can be predicted from first principles. Assuming a random variable, *X*, its variance can be calculated as *E*[*X* ^2^]–*E*[*X*]^2^, where *E* is the expected value. Whenever *X* is small, *E*[*X* ^2^] is negligible, then |Var[*X*]|~|*E*[*X*]^2^|, as our results suggest. What makes this particular relationship important in this context is the understanding we can gain about the association between rates of phenotypic and population change given the power law. The fact that the Taylor power law for population and phenotypic rates shown in Fig. 3 is shared (i.e., they have the same slope and intercept) implies a specific relationship between mean rates of phenotypic and population change. This can be shown as follows. First, we can describe the population and phenotypic power laws mathematically as:

(4) $$\text{Var}\left(\frac{1}{N}\frac{dN}{dt}\right)=\alpha_{1}{〈\frac{1}{N}\frac{dN}{dt}〉}^{\beta_{1}}$$

(5) $$\text{Var}\left(\frac{1}{z}\frac{dz}{dt}\right)=\alpha_{2}{〈\frac{1}{z}\frac{dz}{dt}〉}^{\beta_{2}},$$

where $〈\frac{1}{N}\frac{dN}{dt}〉$ and $〈\frac{1}{z}\frac{dz}{dt}〉$ are the mean population and phenotypic rates respectively, α_1_ and α_2_ are intercepts of the power law in log scales, and β_1_ and β_2_ are the scaling parameters. From Fig. 3, we can see that α_1_ ≈ α_2_ and β_1_ ≈ β_2_ ≈ 2. By dividing both sides of eqn (2) by $\text{Var}\left(\frac{1}{N}\frac{dN}{dt}\right)$ and rearranging, we obtain $\frac{\text{Var}\left(\frac{1dz}{zdt}\right)}{\text{Var}\left(\frac{1dN}{Ndt}\right)}=\frac{\alpha_{2}{〈\frac{1dz}{zdt}〉}^{2}}{\alpha_{1}{〈\frac{1dN}{Ndt}〉}^{2}}.$ Because α_1_≈α_2_, they are canceled, and therefore, this can be further rearranged into the following:

(6) $$〈\frac{1dz}{zdt}〉=\sqrt{\frac{\text{Var}\left(\frac{1dz}{zdt}\right)}{\text{Var}\left(\frac{1dN}{Ndt}\right)}}\;\;〈\frac{1}{N}\frac{dN}{dt}〉,$$

which links the rate of phenotypic change to the rate of change in population abundance through the term in the radical. Note that eqn (6) has the same structure as eqn (3). Because $\text{Var}\frac{1}{N}\frac{dN}{dt}$ $\text{Var}\frac{1}{z}\frac{dz}{dt}$ (Fig. 3), the value in the radical in eqn (6) is <1, suggesting that the mean phenotypic rate of change will generally be smaller than the average rate of population change, as shown empirically in Fig. 1. Because the current observations are also likely biased toward systems with rapid evolution, since such systems are obviously more attractive for the study of evolution, we suggest that the constraint space shown in Fig. 1 is likely to contain most observations from future studies.

In conclusion, although some rates of phenotypic change can be very fast, these rates are on average only about 1/4 of the associated rates of population change and generally not linked to the rate of change in population size. Slower rates of phenotypic than population change may be due to a low fraction of heritable variation or shallow relative fitness gradients, or in some cases a lack of plasticity. Our results may be consistent with the view that evolutionary and ecological time converges (Hairston et al. 2005), but it clarifies that ecological change is mostly much quicker, even when eco‐evolutionary dynamics are important (Grant and Grant 2002; Fussmann et al. 2003).

## Conflict of Interest

None declared.

## Supporting information


**Table S1.** Average (±SE) rate of phenotypic and population size change used in this study.
