# Supplementary material for: How fast is fast? Eco‐evolutionary dynamics and rates of change in populations and phenotypes
Source: Ecol Evol. 2016 Jan 9;6(2):573–81. doi: 10.1002/ece3.1899 (PMC4729258; doi:10.1002/ece3.1899)
Supplement: Supplementary file 1 — Table S1. Average (±SE) rate of phenotypic and population size change used in this study. [file ECE3-6-573-s001.docx]

**Supplemental Material**

**How fast is fast? Eco-evolutionary dynamics and rates of change in populations and phenotypes.**

John P. DeLong, Valery E. Forbes, Nika Galic, Jean P. Gibert, Robert G. Laport, Joseph S. Phillips, Janna M. Vavra

doi: 10.1002/ece3.1899

**Table S1. Average (±SE) rate of phenotypic and population size change used in this study.**

|  |  | **Phenotypic rate** | | **Ecological rate** | |
| --- | --- | --- | --- | --- | --- |
| **Reference** | **Species (& trait if more than one)** | **Mean** | **SE** | **Mean** | **SE** |
| Fussmann et al. 2003 | *Brachionus calyciflorus* | 0.07 | 0.014 | 0.44 | 0.109 |
| DeLong et al. 2014 | *Didinium nasutum* | 0.27 | 0.075 | 0.99 | 0.318 |
| González et al. 1993 | *Cafeteria* sp. | 0.00 | 0.001 | 0.04 | 0.024 |
| Ozgul et al. 2010 | *Marmota flaviventris* | 0.01 | 0.003 | 0.14 | 0.028 |
| Brown and Brown 2013 | *Petrochelidon pyrrhonota* | 0.00 | 0.001 | 0.18 | 0.032 |
| Coltman et al. 2003 | *Ovis Canadensis* (size) | 0.36 | 0.060 | 0.75 | 0.109 |
|  | *Ovis Canadensis* (horns) | 0.45 | 0.075 | 0.75 | 0.109 |
| Milot et al. 2011 | *Homo sapiens* | 0.00 | 0.001 | 1.46 | 0.822 |
| Becks et al. 2012 | *Chlamydomonas reinhardtii* | 0.09 | 0.011 | 0.11 | 0.011 |
| Sinervo et al. 2000 | *Uta stansburiana* (clutch) | 0.09 | 0.017 | 0.49 | 0.110 |
|  | *Uta stansburiana* (egg) | 0.11 | 0.024 | 0.49 | 0.110 |
| Caron et al. 1985 | *Paraphysomonas imperforata* | 0.20 | 0.045 | 0.58 | 0.179 |
| Schoener et al. 2002 | *Anolis sagrei* (hindlimb) | 0.01 | 0.002 | 1.68 | 0.821 |
|  | *Anolis sagrei* (lamellae) | 1.01 | 0.884 | 1.68 | 0.821 |
| Swain et al. 2007 | *Gadus morhua* | 0.16 | 0.022 | 0.89 | 0.164 |
| Ozgul et al. 2009;  Ezard et al. 2009 | *Ovis aries* | 0.03 | 0.007 | 0.23 | 0.044 |
| Grant and Grant 2002 | *Geospiza fortis* (bill depth) | 0.13 | 0.015 | 1.62 | 0.474 |
|  | *Geospiza fortis* (bill length) | 0.08 | 0.019 | 1.62 | 0.474 |
| Fenchel and Jonsson 1988 | *Strombidium sulcatum* | 0.33 | 0.092 | 0.55 | 0.288 |
| Galliard et al. 2005 | *Zootoca oviparis* | 0.47 |  | 1.85 |  |
| Edeline et al. 2008 | *Perca fluviatilis* | 0.19 | 0.012 | 5.90 | 1.274 |

**References**

Becks, L., S. P. Ellner, L. E. Jones, and N. G. Hairston Jr. 2012. The functional genomics of an eco-evolutionary feedback loop: linking gene expression, trait evolution, and community dynamics. Ecology letters 15:492–501.

Brown, C. R., and M. B. Brown. 2013. Where has all the road kill gone? Current Biology 23:R233–R234.

Caron, D. A., J. C. Goldman, O. K. Andersen, and M. R. Dennett. 1985. Nutrient cycling in a microflagellate food chain: II. Population dynamics and carbon cycling. Marine Ecology Progress Series 24:243–254.

Coltman, D. W., P. O’Donoghue, J. T. Jorgenson, J. T. Hogg, C. Strobeck, and M. Festa-Bianchet. 2003. Undesirable evolutionary consequences of trophy hunting. Nature 426:655–658.

DeLong, J. P., T. C. Hanley, and D. A. Vasseur. 2014. Predator–prey dynamics and the plasticity of predator body size. Functional Ecology 28:487–493.

Edeline, E., T. B. Ari, L. A. Vøllestad, I. J. Winfield, J. M. Fletcher, J. B. James, and N. C. Stenseth. 2008. Antagonistic selection from predators and pathogens alters food-web structure. Proceedings of the National Academy of Sciences 105:19792–19796.

Ezard, T. H. G., S. D. Côté, and F. Pelletier. 2009. Eco-evolutionary dynamics: disentangling phenotypic, environmental and population fluctuations. Philosophical Transactions of the Royal Society B: Biological Sciences 364:1491–1498.

Fenchel, T., and P. R. Jonsson. 1988. The functional biology of *Strombidium sulcatum*, a marine oligotrich ciliate (Ciliophora, Oligotrichina). Marine Ecology Progress Series 48:1–15.

Fussmann, G. F., S. P. Ellner, and N. G. Hairston Jr. 2003. Evolution as a critical component of plankton dynamics. Proceedings of the Royal Society B 270:1015–1022.

Galliard, J.-F. L., P. S. Fitze, R. Ferrière, and J. Clobert. 2005. Sex ratio bias, male aggression, and population collapse in lizards. Proceedings of the National Academy of Sciences of the United States of America 102:18231–18236.

González, J. M., E. B. Sherr, and B. F. Sherr. 1993. Differential feeding by marine flagellates on growing versus starving, and on motile versus nonmotile, bacterial prey. Marine Ecology Progress Series 102:257–267.

Grant, P. R., and B. R. Grant. 2002. Unpredictable evolution in a 30-Year study of Darwin’s finches. Science 296:707–711.

Milot, E., F. M. Mayer, D. H. Nussey, M. Boisvert, F. Pelletier, and D. Réale. 2011. Evidence for evolution in response to natural selection in a contemporary human population. Proceedings of the National Academy of Sciences 108:17040–17045.

Ozgul, A., D. Z. Childs, M. K. Oli, K. B. Armitage, D. T. Blumstein, L. E. Olson, S. Tuljapurkar, and T. Coulson. 2010. Coupled dynamics of body mass and population growth in response to environmental change. Nature 466:482–485.

Ozgul, A., S. Tuljapurkar, T. G. Benton, J. M. Pemberton, T. H. Clutton-Brock, and T. Coulson. 2009. The dynamics of phenotypic change and the shrinking sheep of St. Kilda. Science 325:464–467.

Schoener, T. W., D. A. Spiller, and J. B. Losos. 2002. Predation on a common *Anolis* lizard: Can the food-web effects of a devastating predator be reversed? Ecological Monographs 72:383–407.

Sinervo, B., E. Svensson, and T. Comendant. 2000. Density cycles and an offspring quantity and quality game driven by natural selection. Nature 406:985–988.

Swain, D. P., A. F. Sinclair, and J. Mark Hanson. 2007. Evolutionary response to size-selective mortality in an exploited fish population. Proceedings of the Royal Society B 274:1015–1022.
